# Interleukin-2-Inducible T-Cell Kinase Deficiency Impairs Early Pulmonary Protection Against *Mycobacterium tuberculosis* Infection

**DOI:** 10.3389/fimmu.2019.03103

**Published:** 2020-01-24

**Authors:** Lu Huang, Kaixiong Ye, Michael C. McGee, Natalie F. Nidetz, Jessica P. Elmore, Candice B. Limper, Teresa L. Southard, David G. Russell, Avery August, Weishan Huang

**Affiliations:** ^1^Department of Microbiology and Immunology, College of Veterinary Medicine, Cornell University, Ithaca, NY, United States; ^2^Department of Genetics, University of Georgia, Athens, GA, United States; ^3^Institute of Bioinformatics, University of Georgia, Athens, GA, United States; ^4^Department of Pathobiological Sciences, School of Veterinary Medicine, Louisiana State University, Baton Rouge, LA, United States; ^5^Department of Biomedical Sciences, College of Veterinary Medicine, Cornell University, Ithaca, NY, United States

**Keywords:** active tuberculosis, transcriptomic analysis, non-receptor tyrosine kinase, IL-17A, IFN-γ, γδ T cells

## Abstract

Interleukin-2 (IL-2) inducible T-cell kinase (ITK) is a non-receptor tyrosine kinase highly expressed in T-cell lineages and regulates multiple aspects of T-cell development and function, mainly through its function downstream of the T-cell receptor. *Itk* deficiency can lead to CD4 lymphopenia and Epstein-Bar virus (EBV)-associated lymphoproliferation and recurrent pulmonary infections in humans. However, the role of the ITK signaling pathway in pulmonary responses in active tuberculosis due to *Mtb* infection is not known. We show here that human lungs with active tuberculosis exhibit altered T-cell receptor/ITK signaling and that *Itk* deficiency impaired early protection against *Mtb* in mice, accompanied by defective development of IL-17A-producing γδ T cells in the lungs. These findings have important implications of human genetics associated with susceptibility to *Mtb* due to altered immune responses and molecular signals modulating host immunity that controls *Mtb* activity. Enhancing ITK signaling pathways may be an alternative strategy to target *Mtb* infection, especially in cases with highly virulent strains in which IL-17A plays an essential protective role.

## Introduction

*Mycobacterium tuberculosis* (*Mtb*) is the causative agent of what remains one of the most insidious and invasive human infections, responsible for millions of cases of active lung diseases and deaths per year in the world [WHO Global Tuberculosis Report, (1) and O'Garra et al. (2)]. The immune responses by macrophages, neutrophils, and T-cell populations contribute to protective immunity controlling *Mtb* expansion in the host and transmission to others (2, 3). Genetic and environmental factors of the host associated with primary and acquired immunodeficiency can lead to an increased risk of developing active tuberculosis that presents severe pulmonary illness in the clinic (2, 4). Our knowledge of the molecular pathways of innate and adaptive immune effector functions remains incomplete, and a better understanding of potential host factors underlying the pulmonary complications could lead to the development of more innovative therapeutic strategies.

Interleukin-2 (IL-2)-inducible T-cell kinase (ITK) is a non-receptor tyrosine kinase highly expressed in T cells. ITK functions downstream of the T-cell receptor (TCR) and regulates multiple aspects of T-cell development and function (5). *ITK* deficiency in humans is associated with primary immunodeficiency, progressive natural killer T (NKT) and CD4^+^ T-cell lymphopenia, elevated susceptibility to Epstein-Bar virus (EBV), and EBV-driven lymphoproliferative diseases, in which frequent pulmonary involvement has emerged as a clinical hallmark (6–13). Recurrent progressive pulmonary infection, airway obstruction, and respiratory failure in *ITK*-deficient patients pose significant threats that can eventually result in deaths of the patients at their early ages (12). Human T cells from patients with *ITK* deficiency exhibit impaired responses to TCR activation, with reduced generation of Th17 cells and production of the associated cytokines IL-17A, IL-22, and granulocyte-macrophage colony-stimulating factor (14). A murine model of *Itk* deficiency reveals a similar NKT and T-cell lymphopenia as observed in human patients with *ITK* deficiency. Mice lacking *Itk* have a marked reduction in NKT cells (15–18). Despite relatively normal number (trending the lower range) of CD8^+^ αβ T cells, *Itk*^−/−^ mice exhibited CD4^+^ αβ T-cell lymphopenia, with reduced proportion of naive and increased memory αβ T cells (19–31). In the absence of *Itk*, mouse CD4^+^ T cells are impaired in Th2 (producing IL-4/5/13) (32–38), Th9 (producing IL-9) (39), Th17 (producing IL-17) (35, 40–42), and Tr1 (producing IL-10) cell responses (43), while they are enhanced in Th1 (producing IFN-γ) cell response (32, 34, 38, 44, 45). Analysis of *Itk*^−/−^ mice also reveals altered γδ T-cell development (46–49); however, the presence and function of γδ T cells has not been evaluated in *ITK*-deficient humans.

Epidemiological analysis of single-nucleotide polymorphism has also revealed a connection between greater *ITK* promoter activity and higher risk of asthmatic incidence in humans, which might be associated with the function of ITK in promoting T-cell activation (50). In murine models of allergic asthma, the expression of ITK is critical for the activation and development of Th2 and Th17 cells and the associated airway and tracheal inflammation (40, 51). Interestingly, a genome-wide association study of susceptibility to *Mycobacterium avium* subspecies *paratuberculosis* in Holstein cattle identified chromosomal regions that included the *ITK* gene (52). However, the role of ITK signaling pathway in pulmonary responses in active tuberculosis due to *Mtb* infection is unknown.

Here, we show that the TCR/ITK signaling pathway is enriched in human lungs with active tuberculosis and that *Itk* deficiency impaired early protection against *Mtb* in mice, accompanied by defective development of IL-17A-producing γδ T cells in the lungs. Furthermore, ITK appears to regulate the dynamics of lung myeloid cells, which may further contribute to immune control of *Mtb* at the early stage of infection.

## Materials and Methods

### Mice

All mice were on the C57BL/6 background. Both female and male mice at the age of 6–12 weeks were used. All experiments were approved by the Office of Research Protections Institutional Animal Care and Use Committee at Cornell University.

### Microarray and Data Analyses

Microarray data from lung normal tissue and caseous granulomas from active tuberculosis (TB) patient was generated as previously described (53, 54). Microarray data is available in the Gene Expression Omnibus under accession number GSE20050. In brief, tissues were fixed, and areas of interest were dissected using laser capture microdissection on the Leica AS LMD system (Leica, Buffalo Grove, IL). Total RNA was isolated and used on the GeneChip Human X3P Array (Affymetrix, Santa Clare, CA) following the manufacturer's instruction. Data analysis was performed in R (version 3.5.3) and Bioconductor (version 3.8). Probe intensities were log_2_ transformed and median centered. Differentially expressed genes were identified with limma (version 3.38.3) (55). In the case of multiple probes mapped to a gene, the probe with the maximum fold change was selected to represent the gene. Gene set enrichment analyses (GSEA) to determine over- and underrepresented gene sets were performed using the Kyoto Encyclopedia of Genes and Genomes pathway database as reference in gage (version 2.32.1) (56). All pathways that exhibited an up- or downregulated trend in caseum samples compared to normal tissues are summarized in the Supplementary Material (Supplementary Table 1). Enrichment score and core genes that drive the score are determined using the GSEA platform developed by the Broad Institute (57). Visualization of TCR signaling pathway with differential gene expression was performed in pathview (version 1.22.3) (Supplementary Figure 1) (58).

### *Mtb* Infection and Colony-Forming Unit Counts

Mice were inoculated intranasally with ~1,000 CFUs of Erdman *Mtb* constitutively expressing mCherry (mCherry-*Mtb*) (59) in 25 μl of phosphate-buffered saline (PBS) containing 0.05% Tween-80. Mice were euthanized after 2 and 4 weeks of infection. The left lung lobe and the accessory lobe of the right lung were removed and homogenized in PBS containing 0.05% Tween-80. Bacterial loads were determined by plating serial dilutions of the homogenates on 7H10 agar plates.

### Histology and Pathogenic Scoring

Lung samples were fixed in 4% paraformaldehyde overnight, followed by hematoxylin and eosin (H&E) staining. Histological images were analyzed using DP2-BSW software (Olympus, Waltham, MA) to quantify the percentage of affected tissue area and score the severity of pathology (60).

### Isolation of Lung Cells

Mice were euthanized at the indicated time points, and lungs were aseptically removed. To obtain a single-cell suspension, lungs were minced and digested in 5% fetal bovine serum/PBS solution containing 250 U/ml collagenase IV (Worthington, Lakewood, NJ) and 20 U/ml DNase (Roche, Indianapolis, IN) for 30 min at 37°C. Lung digestions were then passed through a 70-μm cell strainer, and red blood cells were lysed with ammonium-chloride-potassium buffer.

### Fluorescent Mouse Antibodies

Fluorescent antibodies are listed in the format of “Fluorophore-target (clone)”: eFluor 450-CD4 (GK1.5), Phycoerythrin (PE)-Foxp3 (FJK-16s), PE-eFluor 610-NK1.1 (PK136), allophycocyanin (APC)-IL-17A (eBio17B7), APC-CD11c (N418), PerCP-eFluor 710-CD49b (DX5), PE-Cy7-NK1.1 (PK136), PE-Cy7-IFN-γ (XMG1.2), and APC-eFluor 780-MHCII (M5/114.15.2) were from eBioscience (San Diego, CA). CD16/32 (93; i.e., Fc block), Brilliant Violet 421-CD64 (X54-5/7.1), Alexa Fluor 488-TCRγδ (GL3), and APC-Cy7-TCRβ (H57-597) were from BioLegend (San Diego, CA). FITC-Ly6G (1A8), PE-Siglec-F (E50-2440), PE-TNF-α (MP6-XT22), PE-CF594 CD8α (53-6.7), Alexa Fluor 700-Ki67 (B56), PerCP-Cy5.5-CD8α (53-6.7), and PerCP-Cy5.5-CD11b (M1/70) were from BD Biosciences (San Jose, CA).

### T-Cell Stimulation

To activate bulk T cells, cells were stimulated with phorbol 12-myristate 13-acetate (PMA) (50 ng/ml, Sigma) and Ionomycin (0.5 μM, Sigma); to activate *Mtb*-specific CD4^+^ T cells, cells were stimulated with ESAT-6_4−17_ peptide (MHCII-restricted presentation; synthesized by GenScript, purity > 95%; 5 μg/ml); to activate *Mtb*-specific CD8^+^ T cells, cells were stimulated with TB10.4_4−11_ peptide (MHCI-restricted presentation; synthesized by GenScript, purity > 95%; 5 μg/ml). All stimulations were done in full RPMI-1640 media in the presence of Brefeldin A (5 μg/ml, Sigma) and Monensin (2 μM, Sigma), at 37°C for 5 h.

### Flow Cytometry

Surface protein staining was done with antibodies for surface markers in PBS, in the presence of Fc Block (BioLegend) and fixable viability dye (Tonbo Biosciences). To determine cytokine production, cells were stimulated as indicated, followed by surface staining, then were fixed with 4% paraformaldehyde (Electron Microscopy Sciences, Hatfield, PA), and permeabilized and stained with antibodies in PBS containing 0.3% saponin (Sigma). To stain for nuclear transcription factors Foxp3 and Ki67, following surface staining, cells were fixed, permeabilized, and stained using Foxp3 staining buffer set (eBioscience). All flow cytometry data were acquired on LSRII (BD Biosciences) and analyzed in FlowJo (Tree Star, Ashland, OR).

### Statistical Analysis

Two-tailed Student's *t*-test and two-way analysis of variance (ANOVA) between groups were performed using Prism (GraphPad, San Diego, CA), with *p* < 0.05 considered statistically significant. “NS” indicates differences that are not significant.

## Results

### TCR/ITK Signaling Components Are Upregulated in Active Tuberculosis in Humans

**Figure 1:**
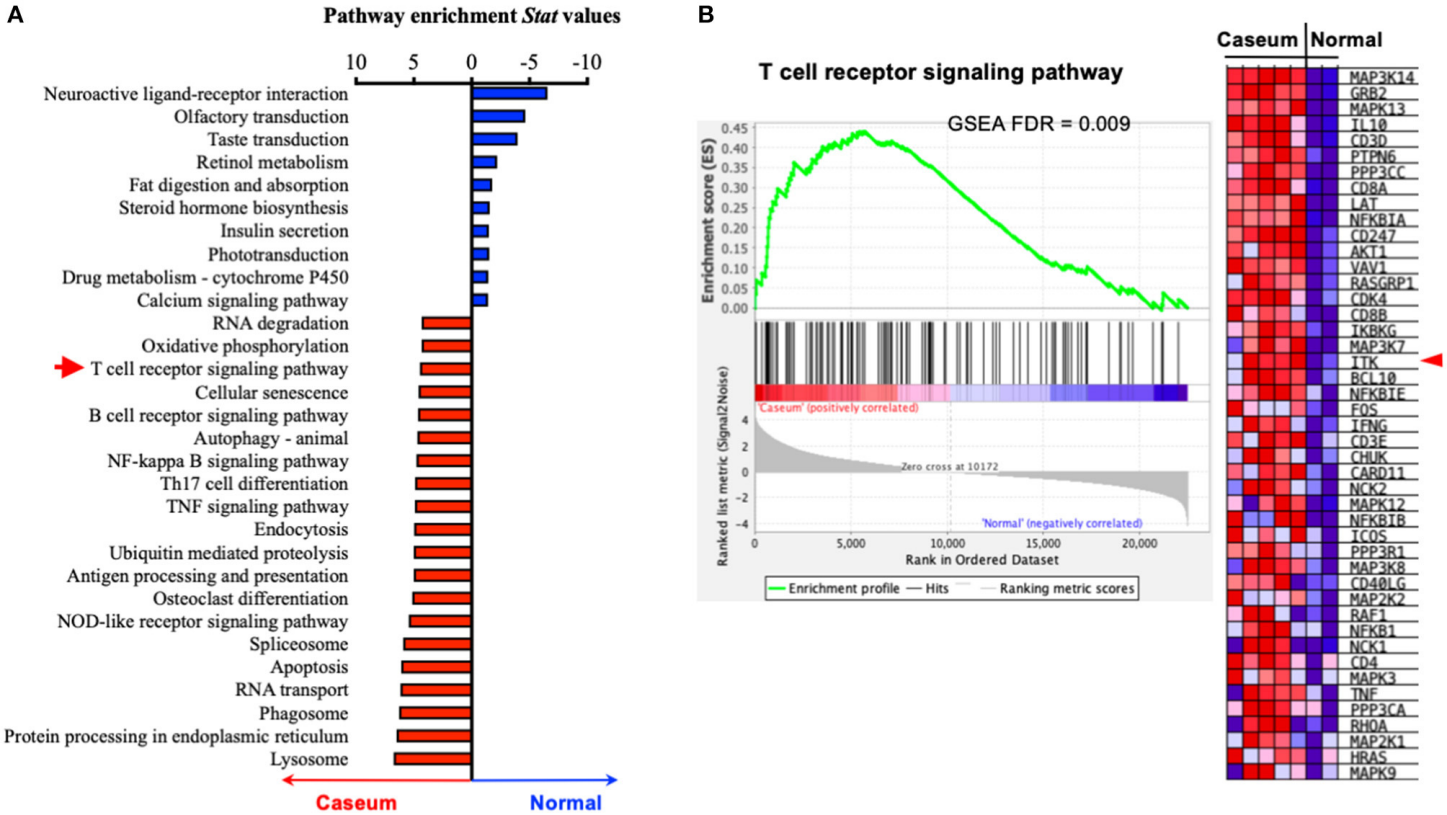
ITK expression is altered in human lungs with active TB. **(A)** Pathway enrichment analyses of human lung tissues with active TB (“Caseum,” *N* = 5), compared to normal lung tissues (“Normal,” *N* = 2). Pathway enrichment statistical values were for pathways enriched in “Caseum” group were shown as positive values (upregulated in “Caseum” compared to “Normal”), while those enriched in normal lungs were shown as negative values (upregulated in “Normal” compared to “Caseum”). Twenty most enriched pathways in the “Caseum” group (out of 145 pathways with FDR < 0.05), and 10 most enriched pathways in “Normal” group (out of 11 pathways with FDR < 0.05) are shown. Red arrow indicates T-cell receptor signaling pathway. **(B)** Gene set enrichment score, profile of T-cell signaling receptor pathway and heatmap of the levels of expression of the score-driving genes in T-cell receptor signaling pathway. Red arrow indicates ITK. FDR, false discover rate.

The progression of human active TB disease and transmission involves the development of the caseous granuloma, in which both *Mtb* and the immune response are active (53). We have previously isolated human granulomata from patients with active TB (Caseum) and analyzed the transcriptomic profile in comparison to uninvolved lung tissue (normal) (53). Using pathway enrichment analyses, we found that genes of the TCR signaling were significantly enriched in the caseum tissue that was subjected to active TB, compared to uninvolved lung tissue (Figure 1). Among the enrichment score-driving critical genes of the TCR signaling, the levels of transcripts for ITK and its signaling components (5) such as LCK, GRB2, SLP76, NCK1, FYN, and PLCG are significantly upregulated in caseated granulomas compared to uninvolved lung tissue (Figure 1B). Among the genes that are significantly enriched in active TB, ITK locates in the hub of the TCR signaling pathway (Supplementary Figure 1). These data imply a role for ITK signaling in host immune activity during active TB.

### *Itk* Deficiency Results in Impaired *Mtb* Clearance and Increased Lung Pathology

**Figure 2:**
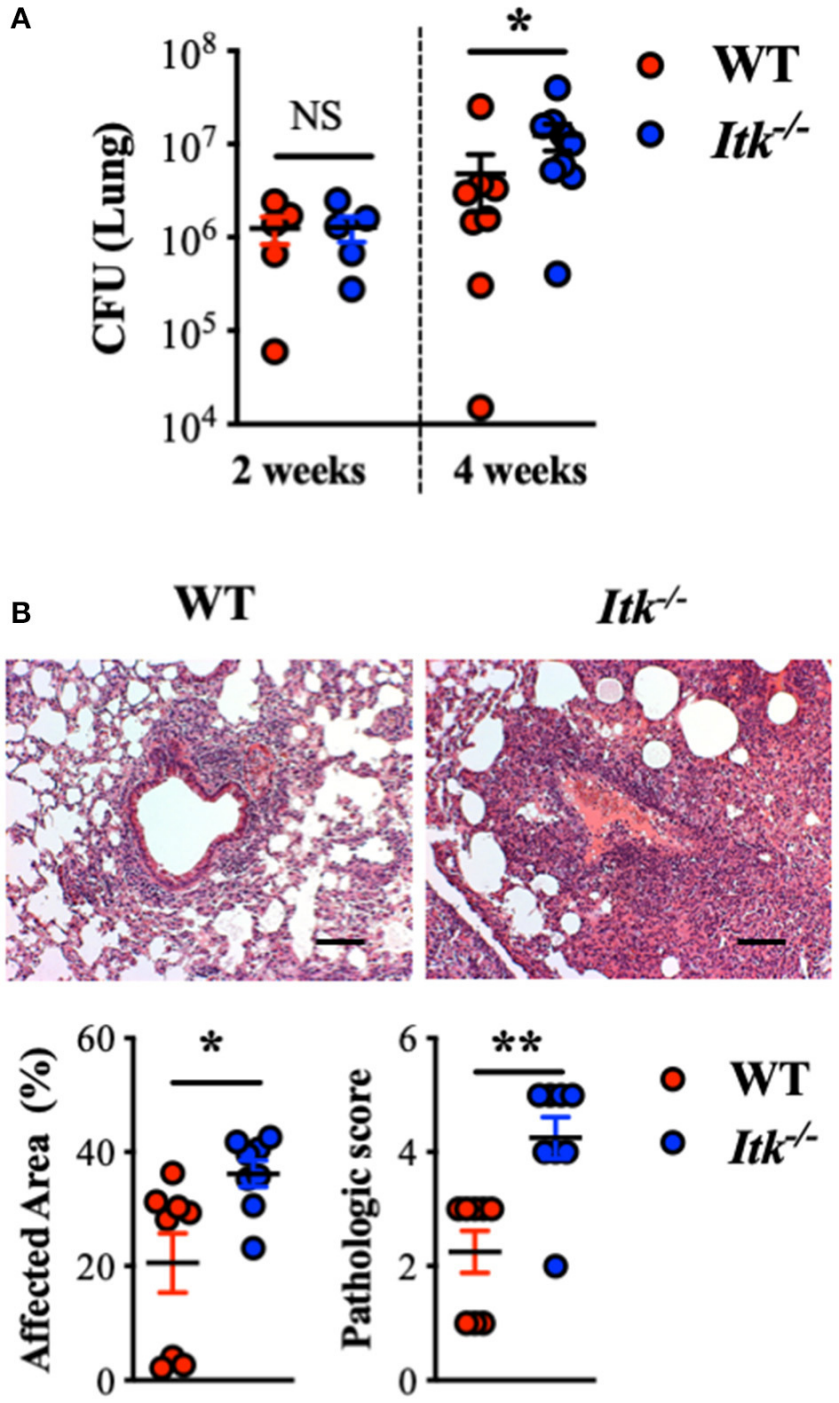
*Itk* deficiency results in impaired bacterial clearance and increased pulmonary pathology post-*Mtb* infection in mice. WT and *Itk*^−/−^ mice were infected with 10^3^ CFU of *Mtb* (intranasal delivery) and analyzed 2 and 4 weeks postinfection. **(A)** CFU in WT and *Itk*^−/−^ mice 2 and 4 weeks postinfection. **(B)** Representative histology, summary of affected area, and histological scoring of WT and *Itk*^−/−^ lungs of mice 4 weeks post-*Mtb* infection. *p < 0.05, **p < 0.01 by Student's *t*-test. Scale bar = 100 μm. *N* = 5–10. Data were presented as mean ± SEM and represent results of three independent experiments.

Given the observation that the TCR signaling pathway was upregulated in the face of active TB in human lungs and that ITK is a critical score-driving gene for the pathway enrichment (Figure 1), we sought to determine the role of ITK in host responses to *Mtb* infection. In murine models of *Itk* deficiency, despite no difference in animal mortality, we found that *Mtb* bacterial burden was significantly higher in the lungs in the absence of ITK 4 weeks post-*Mtb* infection (Figure 2A). Moreover, pulmonary pathology was elevated in the absence of ITK, with significantly larger areas in the airway affected at higher pathological scores (Figure 2B). Notably, compared to wild-type (WT) mice, the relative kinetics of bacterial growth in the lungs of *Itk*^−/−^ and *Rag*^−/−^ mice are similar (61, 62). These data suggest that the TCR/ITK signaling pathway regulates immune responses that contribute to limiting *Mtb* growth and controlling pulmonary inflammation 4 weeks postinfection.

### *Itk*-Deficient Mice Exhibit Altered Early Immune Responses to *Mtb* Infection

**Figure 3:**
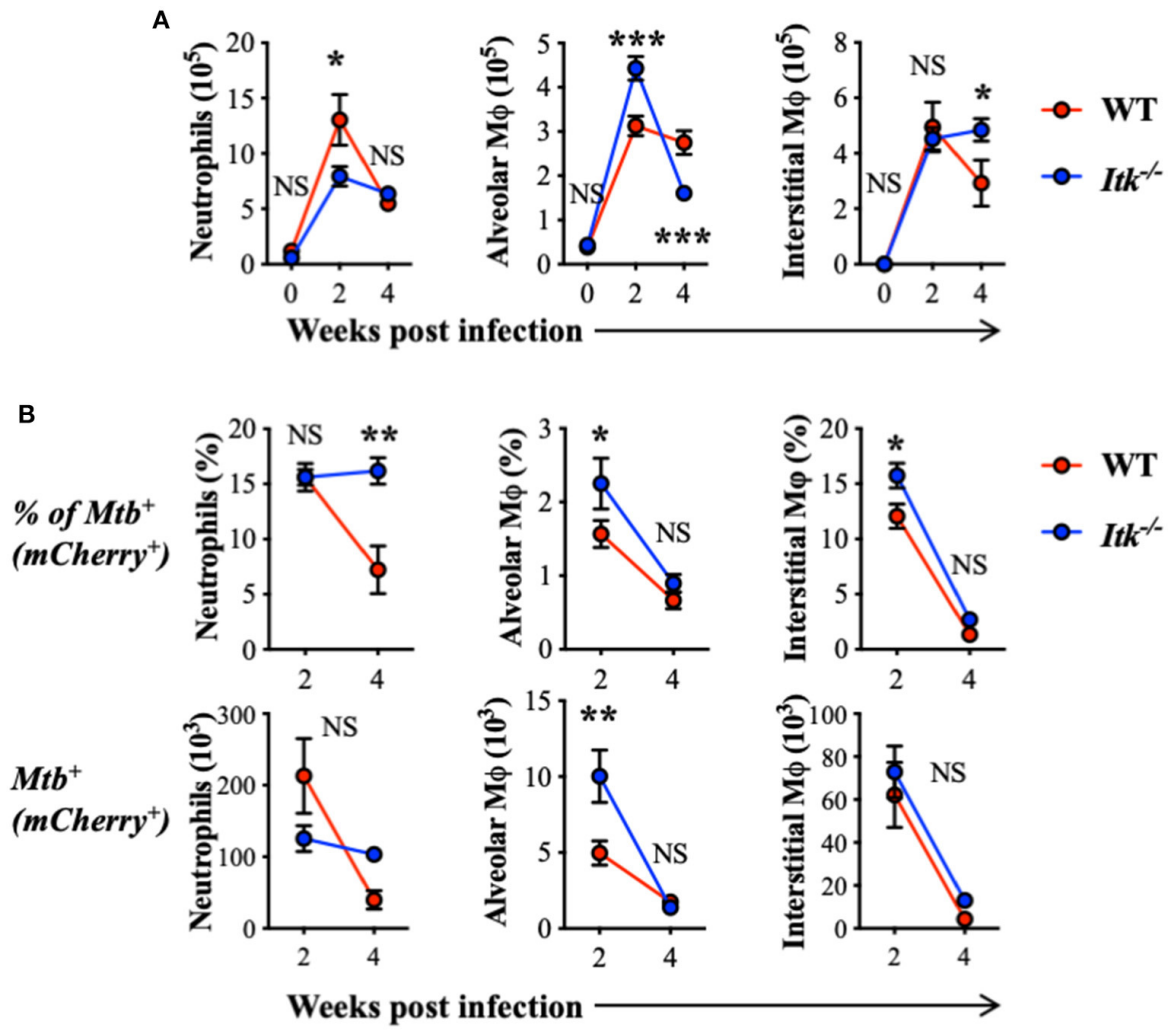
ITK regulates innate myeloid immune response in mice during *Mtb* infection. WT and *Itk*^−/−^ mice were infected with 10^3^ CFU of mCherry-*Mtb*, and lungs were analyzed at the indicated time points. After gating on viable singlet cells, neutrophils (Ly6G^+^CD11b^+^), alveolar macrophages (Ly6G^−^CD11c^+^Siglec-F^+^), and interstitial macrophages (Ly6G^−^Siglec-F^−^ CD11b^+^MHCII^+^CD64^+^) were analyzed. **(A)** Number of total neutrophils, alveolar macrophages, and interstitial macrophages isolated from the lungs of infected mice. **(B)** Percentage and number of mCherry-*Mtb* positive neutrophils, alveolar macrophages, and interstitial macrophages in the lung of the infected mice. NS, not significant; *p < 0.05, **p < 0.01, ***p < 0.001 by two-way ANOVA with *post hoc* test. *N* = 5. Data were presented as mean ± SEM and represent results of three independent experiments.

The progression of human active TB disease and transmission involves the development of both innate and adaptive immunity. The relative abundance of lung phagocyte populations is extremely dynamic at the early stage of *Mtb* infection (63). Importantly, the various phagocytes in the lung provide different environments for *Mtb* and reveal distinct permissiveness for the growth of *Mtb* (64, 65). We thus analyzed populations of innate immune cells in both WT and *Itk*-deficient mice infected with *Mtb* that constitutively express fluorescent protein mCherry, which allows mapping of the cellular location of intracellular bacteria. During early-stage infection, we observed increased total numbers of neutrophils and alveolar macrophages in the lung at 2 weeks postinfection in both WT and *Itk*^−/−^ mice (Figure 3A). Further analysis of the pulmonary immune cell populations revealed that, in the absence of ITK, the proportion of *Mtb*-infected alveolar and interstitial macrophages was significantly higher early after infection (2 weeks); in addition, the proportion of *Mtb*-infected neutrophils was significantly higher 4 weeks after infection (Figure 3). Effective removal of *Mtb*-infected apoptotic neutrophils by macrophages, or efferocytosis, is considered beneficial for host defense (66). The failed clearance of infected neutrophils in *Itk*-deficient mice at 4 weeks suggests that Itk may be involved in regulating efferocytosis of lung macrophages. Both alveolar macrophages and neutrophils have been demonstrated as permissive cell types in *Mtb* infection by providing a hospitable environment for optimal bacterial growth (64, 65). The increased proportion of *Mtb*-infected neutrophils in the absence of ITK is associated with the increased bacterial burden at 4 weeks, suggesting that bacterial burden may be attributed to this difference. Therefore, these data suggest that ITK regulates the dynamic of lung phagocytes and contributes to host protection against *Mtb* infection.

**Figure 4:**
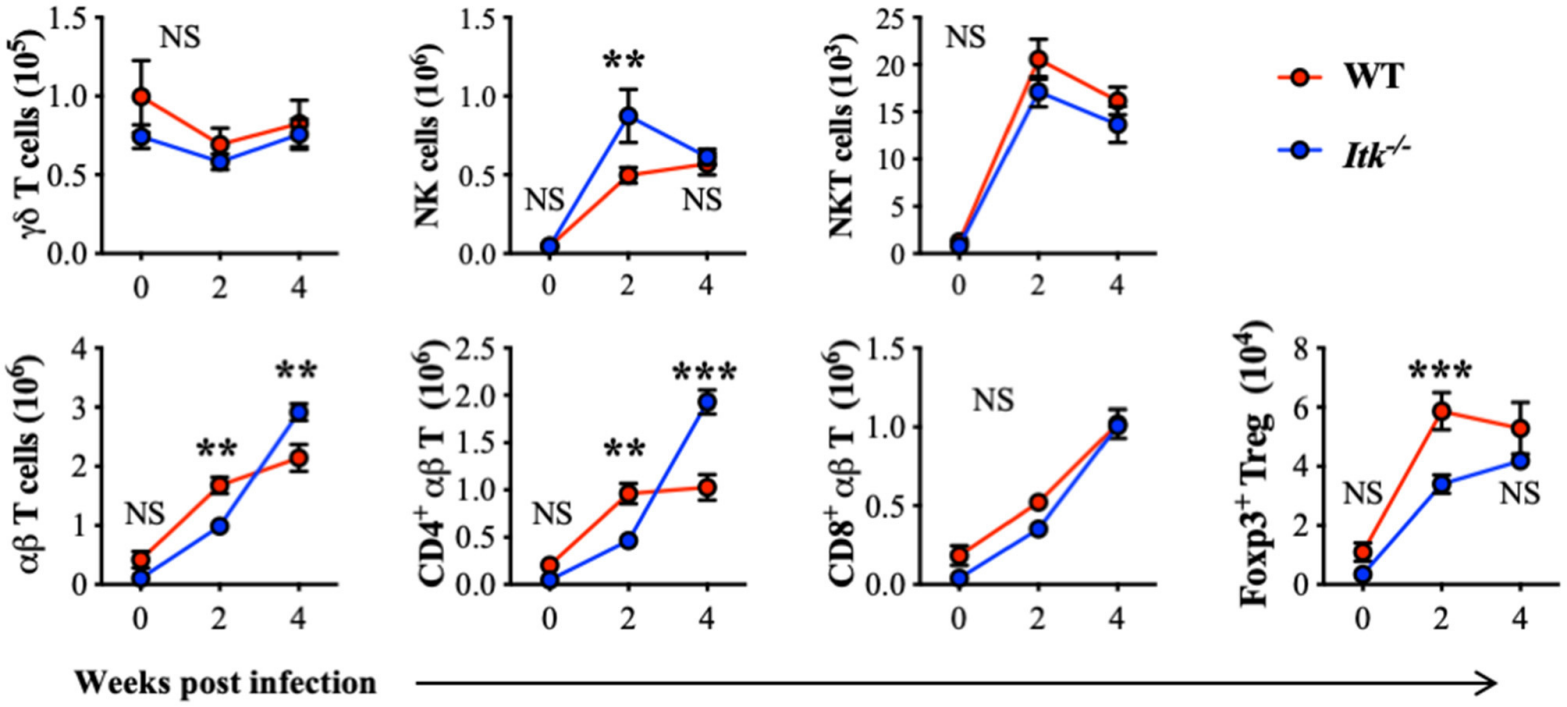
*Itk* deficiency alters lymphocyte profile in the lung during *Mtb* infection. WT and *Itk*^−/−^ mice were infected as in Figure 3, and lungs were analyzed at the indicated time points. After gating on viable singlet cells, lymphocytes including γδT cells (TCRγδ^+^TCRβ^−^), NK cells (TCRγδ^−^TCRβ^−^NK1.1^+^), NKT cells (TCRγδ^−^TCRβ^+^NK1.1^+^), and αβT cells (TCRγδ^−^TCRβ^+^), and among αβT cells, CD4^+^ conventional T cells, CD8^+^ cells, and CD4^+^ Foxp3^+^ Treg cells were analyzed. *N* = 5–9. Numbers of the indicated cell types were presented as mean ± SEM and represent results of three independent experiments. NS, not significant; **p < 0.01, ***p < 0.001 by two-way ANOVA with *post hoc* test.

ITK is highly expressed in T-cell lineages including γδ and αβ T cells. To further determine whether the absence of ITK affects T-cell and other related lymphocyte responses during *Mtb* infection, we also analyzed the abundance of γδ T cells, NK cells, NKT cells, and αβ T cells, including total αβ T cells, CD4^+^ conventional αβ T cells, CD8^+^ αβ T cells, and CD4^+^ Foxp3^+^ regulatory T (Treg) cells. Comparable numbers of γδ T cells, NKT cells, and CD8^+^ αβ T cells were observed in WT and *Itk*-deficient mice infected with *Mtb*, while NK cells and CD4^+^ αβ T cells were significantly increased, and Foxp3^+^ Treg cells were significantly reduced in the absence of ITK (Figure 4). These results seemed surprising, as a reduced number of Treg cells accompanied by increased numbers of CD4^+^ αβ T cells may suggest a more active immune response. The hosts' ability to limit bacterial growth and control pulmonary inflammation was, however, impaired in the absence of ITK. A possible explanation could be that the effector activity of the lymphocytes observed in the infected airway against *Mtb* may differ. These effector immune responses could involve innate lymphocyte activities, as well as *Mtb* antigen-specific adaptive lymphocyte functions.

### ITK Is Critical for γδ T-Cell-Derived IL-17A Production During *Mtb* Infection

**Figure 5:**
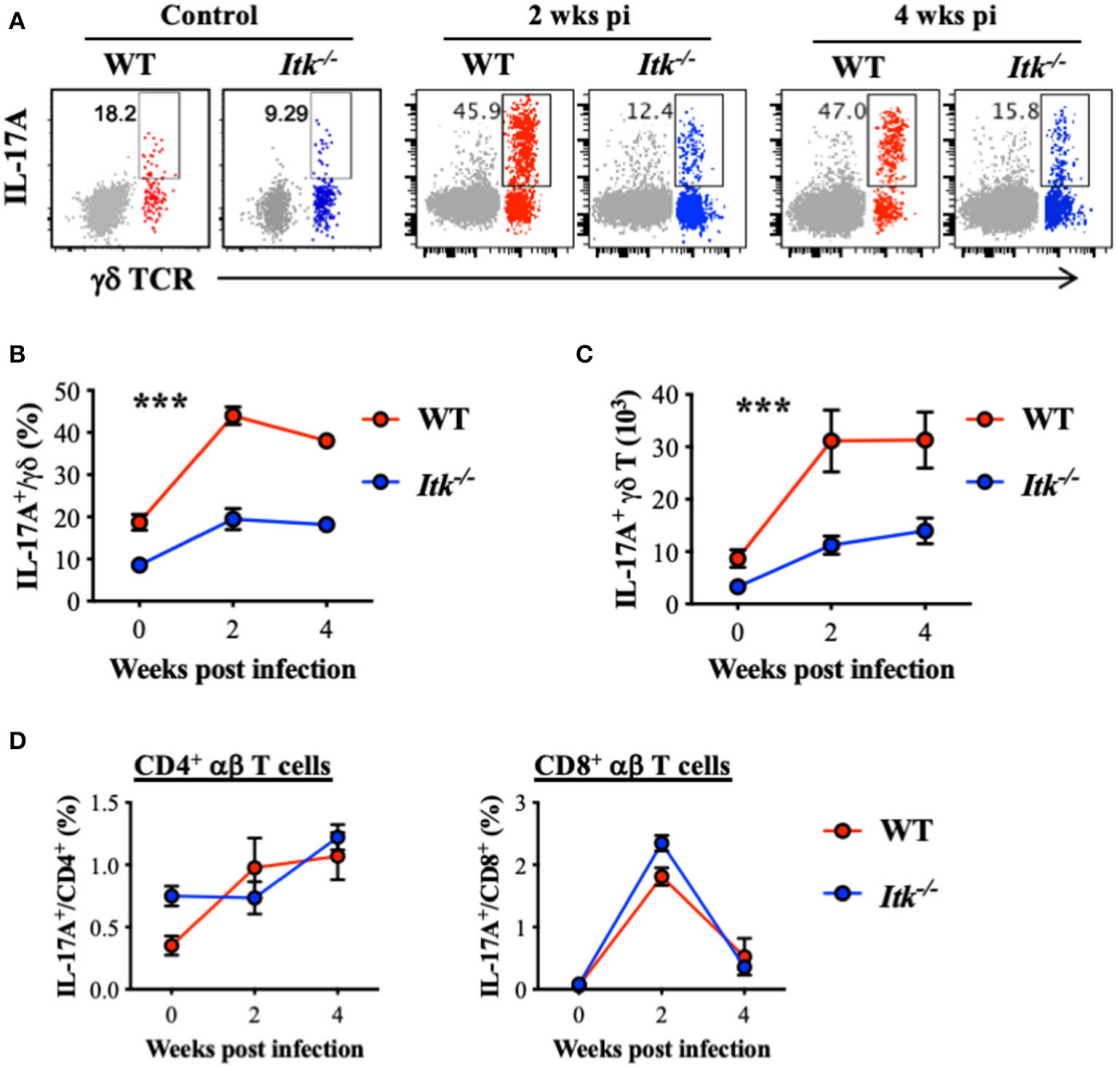
*Itk* deficiency impairs lung γδ T-cell-derived IL-17A production during *Mtb* infection. WT and *Itk*^−/−^ mice were infected as in Figure 3, and lungs were analyzed at the indicated time points. Cells isolated from the lungs were stimulated with PMA and ionomycin, in the presence of BFA and monensin, followed by intracellular cytokine staining. **(A)** Representative FACS plots for IL-17A production by γδ T cells. Production of CD4^+^ αβ T cells is shown as gray background. **(B)** Percentages and **(C)** number of IL-17A-producing γδ T cells. **(D)** Percentages of IL-17A-producing CD4^+^ and CD8^+^ αβ T cells. ***p < 0.001 by two-way ANOVA with *post hoc* test. *N* = 5–9. Numbers of the indicated cell types were presented as mean ± SEM and represent results of three independent experiments.

It has been reported that IL-17A is protective during primary infection of virulent *Mtb* (67), and IL-17A is predominantly produced by γδ T cells in the lungs early after *Mtb* infection (68). Indeed, while we detected significant IL-17A production by γδ T cells in the lungs of WT mice infected with *Mtb* (red foreground in Figures 5A–**C**), very limited IL-17A was produced by the conventional CD4^+^ αβ Th17 cells (gray background in Figure 5A, as well as in Figure 5D). Interestingly, in the absence of ITK, there was a marked reduction in IL-17A-producing γδ T cells in the lung (Figures 5A–C). In contrast to the significant levels of IL-17A production in γδ T cells, IL-17A production by CD4^+^ and CD8^+^ αβ T cells during *Mtb* infection was minimal (Figure 5B vs. Figure 5D). Furthermore, in the absence of ITK, unlike γδ T-cell-derived IL-17A that was significantly impaired, IL-17A production by CD4^+^ and CD8^+^ αβ T cells did not exhibit overt differences, although the proportion of those cells making IL-17A was quite low (Figure 5D).

### *Itk* Deficiency Has Minimal Impact on Antigen-Specific αβ T-Cell Responses During *Mtb* Infection

**Figure 6:**
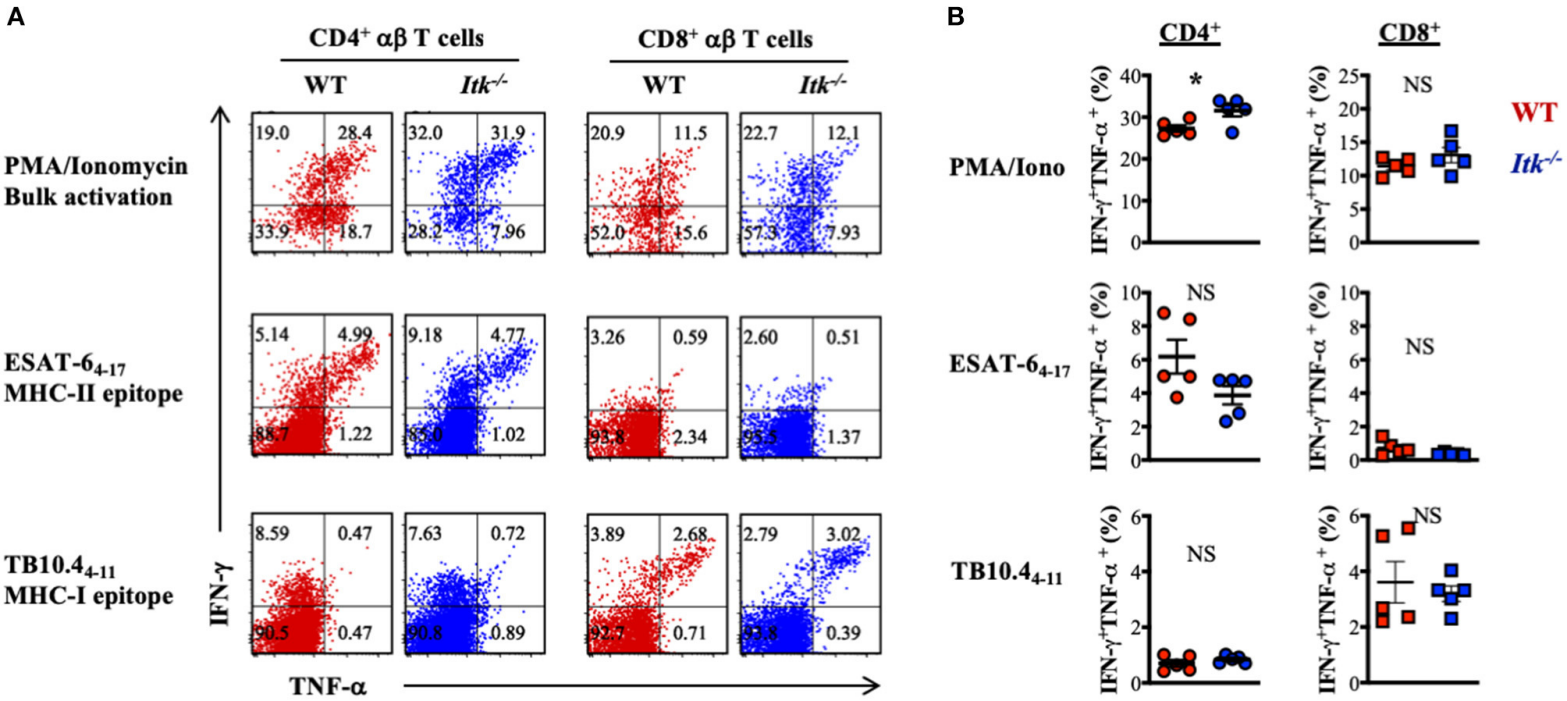
*Itk* deficiency has minimal impact on lung antigen-specific αβ effector T cells during *Mtb* infection. WT and *Itk*^−/−^ mice were infected as in Figure 3 and analyzed 4 weeks postinfection. Cells isolated from the lungs were stimulated with PMA and ionomycin (for bulk T-cell activation), MHCII-restricted *Mtb* epitope ESAT-6_4−17_ (for antigen-specific CD4^+^ T-cell activation), or MHCI- restricted *Mtb* epitope TB10.4_4−11_ (for antigen-specific CD8^+^ T-cell activation), in the presence of BFA and monensin, followed by intracellular cytokine staining. **(A)** Representative FACS plots for IFN-γ and TNF-α production by CD4^+^ and CD8^+^ αβ T cells under the indicated stimulating conditions. **(B)** Summary of percentages of IFN-γ and TNF-α-producing CD4^+^ and CD8^+^ αβ T cells under the indicated stimulating conditions. *N* = 5. Data were presented as mean ± SEM and represent results of three independent experiments. NS, not significant; *p < 0.05 by Student's *t*-test.

Our data above supports a strong role of ITK in promoting the IL-17A-producing effector γδ T cells during *Mtb* infection. It is possible that αβ T-cell effector functions were also altered in the absence of ITK and further contributed to the impaired bacterial clearance and enhanced lung pathology as observed in Figure 2. To determine whether ITK regulates αβ T-cell effector function, we stimulated cells isolated from the lungs of the infected mice 4 weeks postinfection. We found that bulk T-cell activation by PMA and ionomycin suggested that ITK is not required for CD4^+^ and CD8^+^ αβ T cells to produce effector cytokines tumor necrosis factor alpha (TNF-α) and interferon-gamma (IFN-γ) during *Mtb* infection (Figure 6A). Moreover, to our surprise, stimulation of *Mtb* antigen-specific CD4^+^ αβ T cells with ESAT-6_4−17_ (MHCII-restricted epitope), or CD8^+^ αβ T cells with TB10.4_4−11_ (MHCI-restricted epitope) revealed no difference in production of TNF-α and IFN-γ by these cells in the absence of ITK (Figure 6B). Along with the data above, our results suggest that the major protective role of ITK during *Mtb* infection might be executed through ITK-mediated IL-17A production by γδ T cells.

### *Itk* Regulates γδ T Cell but Not αβ T-Cell Proliferation During *Mtb* Infection

**Figure 7:**
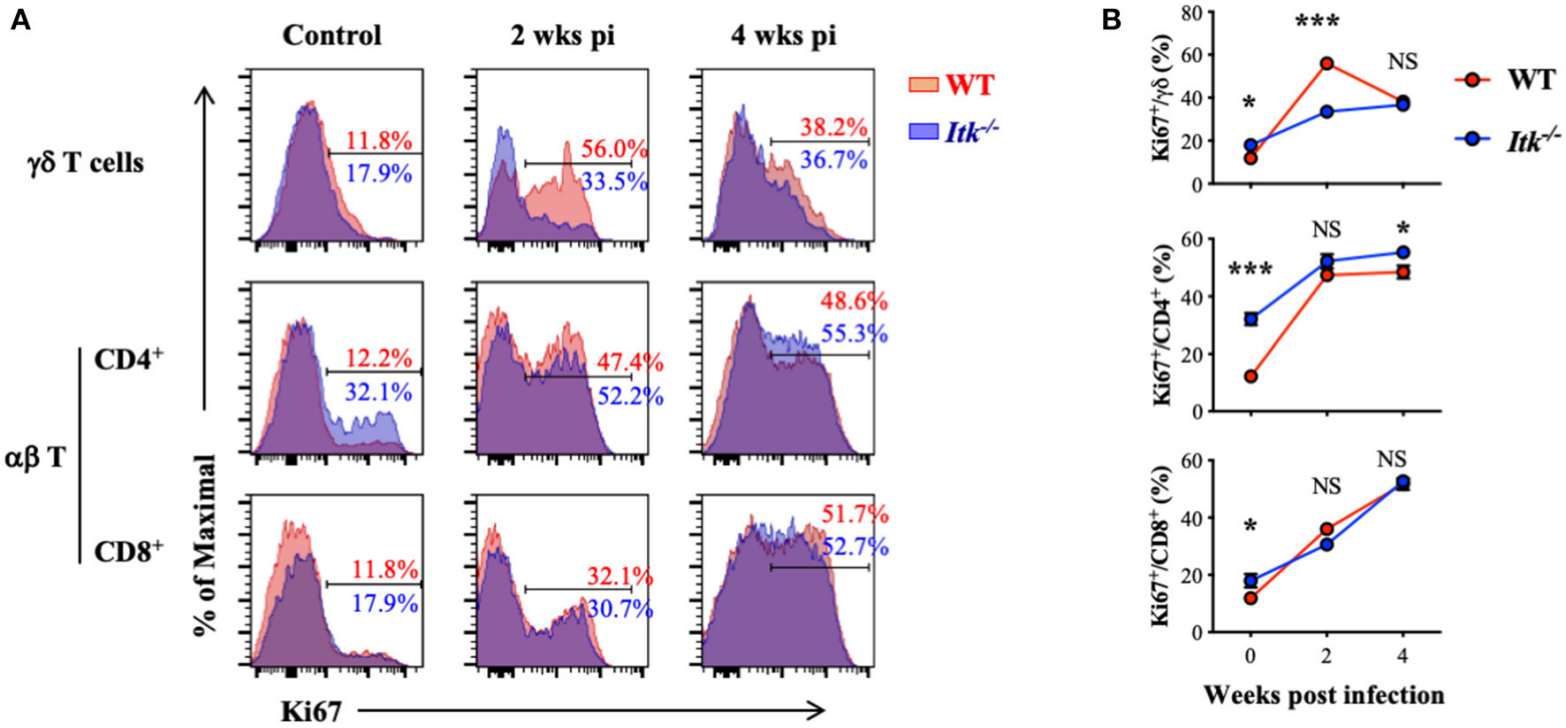
*Itk* deficiency impairs lung γδ but not αβ T-cell proliferation during *Mtb* infection. WT and *Itk*^−/−^ mice were infected as in Figure 3 and analyzed at the indicated time points. Cells isolated from the lungs were analyzed via nuclear staining. **(A)** Representative FACS plots for Ki67 expression by γδ T cells and CD4^+^ and CD8^+^ αβ T cells at the indicated time points postinfection. **(B)** Summary of percentages of KI67^+^ cells among γδ T cells and CD4^+^ and CD8^+^ αβ T cells. *N* = 5–9. Data were presented as mean ± SEM and represent results of three independent experiments. NS, not significant; *p < 0.05, ***p < 0.001 by two-way ANOVA with *post hoc* test.

*Itk* deficiency led to significantly impaired effector γδ T cells (Figure 5) but not αβ T cells (Figure 6). These might be the results of differential requirement of ITK signaling in T-cell expansion. Using proliferative maker Ki67 to detect T-cell proliferation, we found that *Mtb*-driven early proliferation of γδ T cells was severely impaired in *ITK* deficiency (Figure 7, top panel), but not the CD4^+^ and CD8^+^ αβ T-cell subsets (Figure 7, middle and bottom panels). These data, in part, explain the selective defect of effector γδ T-cell development in *Itk*-deficient mice during *Mtb* infection.

## Discussion

The immune cells that control *Mtb* include macrophages, neutrophils, and T-cell populations (2, 3). The TCR pathway is elevated in active *Mtb* infection in human lung, and ITK is a major component of this pathway; however, its role in the T-cell response to *Mtb* is not known. Here, we demonstrate a protective role of ITK in *Mtb* infections in murine models, with a particular role in the ability of lung γδ T cells to produce IL-17A, which is associated with *Mtb* residence in lung neutrophils. These findings have important implications for understanding the T-cell immune response to *Mtb* and the role of ITK in this process.

In humans, *Mtb* infects *via* inhalation of a low dose of aerosolized bacteria; therefore, low-dose aerosol inoculation would better mimic the nature of *Mtb* infection in humans. However, in the absence of the ability to perform aerosolized infections, the standard experimental protocol of *Mtb* infection in mice is 1,000 CFU *via* intranasal inoculation (69–72). Monocytes play an essential role in initiating T-cell responses in the lung against *Mtb* infection (73), and using this standard protocol of *Mtb* infection in mice, we previously observed monocytosis (64), similar to what has been reported in other studies using aerosolized low-dose infection protocols (74, 75). Using intranasal inoculation with 1,000 CFU of *Mtb* in mice, we observed that the numbers of host myeloid cells infiltrating to the lung during *Mtb* infection are significantly altered in the absence of ITK. Alveolar macrophages are considered as the preferred replicating niche for *Mtb* and promote the early stage of infection (64, 76). In *Itk*-deficient mice, there are more alveolar macrophages harboring *Mtb*, suggesting a more permissive cellular environment in the lung. Moreover, although fewer neutrophils are present in the lung in the absence of ITK, there are more *Mtb* resident in neutrophils at 4 weeks postinfection. The redistribution of *Mtb* in different phagocytes in the absence of ITK appears to correlate with the increased bacterial burden in the lung 4 weeks postinfection. Other than T cells, ITK has been indicated to regulate functions of innate immune cells such as mast cells (77, 78). However, potential intrinsic functions of ITK in lung macrophages and neutrophils during *Mtb* infection would merit further studies.

Activation of γδ T cells for IL-17A production is severely impaired in *Itk*-deficient mice and very likely responsible for the resultant neutrophil recruitment early after infection(s), as well as the increased neutrophil resident *Mtb* population later in infection. Indeed, we have shown that ITK can regulate γδ T cell development and function in mouse (48), and while there was no apparent difference in peripheral blood γδ T-cell numbers in humans carrying *ITK* mutations (12), the normal range for γδ T cells varies quite widely by anatomical location, as well as by geography and ethnicity (79). Despite relatively normal antigen-specific αβ T-cell responses, our results identify ITK signaling as an essential player for the IL-17A production by γδ T cells, the predominant source of IL-17A in *Mtb*-infected lungs (68). ITK exhibited a γδ T cell-specific function, as compared to αβ T cells, in driving T-cell proliferation during early immune responses to *Mtb* infections. Our work suggests that *ITK* deficiency in humans may lead to γδ T-cell deficiency in expansion and production of IL-17A, in the face of *Mtb* infection.

We and others have previously reported a role for ITK signaling in regulating Foxp3^+^ regulatory T (Treg) cell development and function (41, 80). In the absence of ITK, the proportion of Treg cells among CD4^+^ T cells in the lymphoid organs of naive mice is increased, and the differentiation of inducible Treg cells from CD4^+^ naive T cells *in vitro* is also enhanced (41, 80). The role of ITK in Treg cell responses in the lung during *Mtb* infection was, however, unclear. In mouse model of *Mtb* infection, we observed higher *Mtb* burdens, higher lung pathological scores and reduced numbers of Treg cells found in the lungs in *Itk*-deficient mice, compared to those in the infected WT mice (Figure 4). Tissue damage may be explained by excessive growth of the pathogen and/or immunopathology. Indeed, we observed impaired γδ T cell expansion and γδ T cell-derived IL-17A production in *Mtb*-infected *Itk*-deficient mice but no overt differences in other immune effectors. The decrease in Treg numbers in the lung during *Mtb* infection in the absence of ITK may explain higher levels of immunopathology potentially due to impaired immunomodulatory function. Future research using murine models with conditional deletion of *Itk* specifically in Foxp3^+^ Treg cells would allow more in-depth investigation of the role of ITK in Treg cells during *Mtb* infection.

Taken together, these findings suggest a potential role for ITK in active TB in humans, in addition to its known connection with primary immunodeficiency, susceptibility to EBV, lymphoproliferative diseases, and asthma (12, 50, 51). These findings also have important implications for human genetics associated with susceptibility to *Mtb* due to altered immune responses and molecular signals modulating host immunity that controls the progression of active tuberculosis.

Our findings support a role of ITK signaling in promoting protective immune responses against *Mtb*, in particular, γδ T cell expansion and production of IL-17A, which could contribute to the modulation of tuberculosis, especially in infections with highly virulent bacterial strains in which IL-17A has been shown to play an essential protective role (67). However, our work also sounds a note of caution for the potential use of compounds such as Ibrutinib that inhibit the related kinase BTK as well as ITK (81). Given the potential for inhibition of ITK, patients being treated with Ibrutinib may need to be monitored for infection or potential reactivation of latent *Mtb*.

## Data Availability Statement

The datasets generated for this study can be found in the Gene Expression Omnibus under accession number GSE20050.

## Ethics Statement

The animal study was reviewed and approved by the Institutional Animal Care and Use Committees at Cornell University.

## Author Contributions

LH, MM, NN, JE, CL, and WH performed the experiments. LH, TS, AA, and WH analyzed and interpreted data. LH, AA, and WH wrote the manuscript. KY performed bioinformatic analyses. DR contributed reagents and intellectual input. AA and WH conceived research and designed experiments.

### Conflict of Interest

AA receives research support from 3M Corporation. The remaining authors declare that the research was conducted in the absence of any commercial or financial relationships that could be construed as a potential conflict of interest.

## Supplementary Material

Supplementary Figure 1 Gene enrichment profile of the TCR/ITK signaling in human lungs with active TB. Genes in the TCR signaling pathways were rendered with their positions and connections in the pathway, with colored codes indicating the log_2_-fold change comparing levels of gene expression in caseous to those in normal tissues. Red indicates upregulation in caseous samples while blue indicates downregulation. Note that ITK is one of the central components of the TCR signaling pathway.

Supplementary Table 1 Summary of pathways that exhibited an up- or down-regulated trend in caseum samples compared to normal tissues.

## Abbreviations

CFU: colony-forming unit
GSEA: gene set enrichment analysis
ITK: IL-2-inducible T-cell kinase
*Mtb*: *Mycobacterium tuberculosis*
NK: natural killer
TCR: T-cell receptor
Treg cells: Foxp3-expressing regulatory T cells.

## References

[1] Global Tuberculosis Report 2019 Geneva: World Health Organization (2019).

[2] O'Garra A Redford PS McNab FW Bloom CI Wilkinson RJ Berry MP. The immune response in tuberculosis. Annu Rev Immunol. (2013) 31:475–527. 10.1146/annurev-immunol-032712-095939 23516984

[3] de Martino M Lodi L Galli L Chiappini E. Immune response to *Mycobacterium tuberculosis*: a narrative review. Front Pediatr. (2019) 7:350. 10.3389/fped.2019.00350 31508399 PMC6718705

[4] Boisson-Dupuis S Bustamante J El-Baghdadi J Camcioglu Y Parvaneh N El Azbaoui S. Inherited and acquired immunodeficiencies underlying tuberculosis in childhood. Immunol Rev. (2015) 264:103–20. 10.1111/imr.12272 25703555 PMC4405179

[5] Andreotti AH Schwartzberg PL Joseph RE Berg LJ. T-cell signaling regulated by the Tec family kinase, Itk. Cold Spring Harb Perspect Biol. (2010) 2:a002287. 10.1101/cshperspect.a002287 20519342 PMC2890196

[6] Tangye SG Palendira U Edwards ES. Human immunity against EBV-lessons from the clinic. J Exp Med. (2017) 214:269–83. 10.1084/jem.20161846 28108590 PMC5294862

[7] Mansouri D Mahdaviani SA Khalilzadeh S Mohajerani SA Hasanzad M Sadr S. IL-2-inducible T-cell kinase deficiency with pulmonary manifestations due to disseminated Epstein-Barr virus infection. Int Arch Allergy Immunol. (2012) 158:418–22. 10.1159/000333472 22487848

[8] Linka RM Risse SL Bienemann K Werner M Linka Y Krux F. Loss-of-function mutations within the IL-2 inducible kinase ITK in patients with EBV-associated lymphoproliferative diseases. Leukemia. (2012) 26:963–71. 10.1038/leu.2011.371 22289921

[9] Huck K Feyen O Niehues T Ruschendorf F Hubner N Laws HJ. Girls homozygous for an IL-2-inducible T cell kinase mutation that leads to protein deficiency develop fatal EBV-associated lymphoproliferation. J Clin Invest. (2009) 119:1350–8. 10.1172/JCI37901 19425169 PMC2673872

[10] Bienemann K Borkhardt A Klapper W Oschlies I. High incidence of Epstein-Barr virus (EBV)-positive Hodgkin lymphoma and Hodgkin lymphoma-like B-cell lymphoproliferations with EBV latency profile 2 in children with interleukin-2-inducible T-cell kinase deficiency. Histopathology. (2015) 67:607–16. 10.1111/his.12677 25728094

[11] Veillette A Perez-Quintero LA Latour S. X-linked lymphoproliferative syndromes and related autosomal recessive disorders. Curr Opin Allergy Clin Immunol. (2013) 13:614–22. 10.1097/ACI.0000000000000008 24113228

[12] Ghosh S Bienemann K Boztug K Borkhardt A. Interleukin-2-inducible T-cell kinase (ITK) deficiency - clinical and molecular aspects. J Clin Immunol. (2014) 34:892–9. 10.1007/s10875-014-0110-8 25339095 PMC4220104

[13] Cohen JI. Primary immunodeficiencies associated with EBV disease. Curr Top Microbiol Immunol. (2015) 390:241–65. 10.1007/978-3-319-22822-8_10 26424649 PMC6349415

[14] Eken A Cansever M Somekh I Mizoguchi Y Zietara N Okus FZ. Genetic deficiency and biochemical inhibition of ITK affect human Th17, Treg, and innate lymphoid cells. J Clin Immunol. (2019) 39:391–400. 10.1007/s10875-019-00632-5 31025232

[15] Qi Q Huang W Bai Y Balmus G Weiss RS August A. A unique role for ITK in survival of invariant NKT cells associated with the p53-dependent pathway in mice. J Immunol. (2012) 188:3611–9. 10.4049/jimmunol.1102475 22403441 PMC3337705

[16] Qi Q Xia M Bai Y Yu S Cantorna M August A. Interleukin-2-inducible T cell kinase (Itk) network edge dependence for the maturation of iNKT cell. J Biol Chem. (2011) 286:138–46. 10.1074/jbc.M110.148205 21036902 PMC3012968

[17] Felices M Berg LJ. The Tec kinases Itk and Rlk regulate NKT cell maturation, cytokine production, and survival. J Immunol. (2008) 180:3007–18. 10.4049/jimmunol.180.5.3007 18292523

[18] Gadue P Stein PL. NK T cell precursors exhibit differential cytokine regulation and require Itk for efficient maturation. J Immunol. (2002) 169:2397–406. 10.4049/jimmunol.169.5.2397 12193707

[19] Liao XC Littman DR. Altered T cell receptor signaling and disrupted T cell development in mice lacking Itk. Immunity. (1995) 3:757–69. 10.1016/1074-7613(95)90065-9 8777721

[20] Bachmann MF Littman DR Liao XC. Antiviral immune responses in Itk-deficient mice. J Virol. (1997) 71:7253–7. 10.1128/JVI.71.10.7253-7257.1997 9311799 PMC192066

[21] Liao XC Littman DR Weiss A. Itk and Fyn make independent contributions to T cell activation. J Exp Med. (1997) 186:2069–73. 10.1084/jem.186.12.2069 9396778 PMC2199174

[22] Schaeffer EM Debnath J Yap G McVicar D Liao XC Littman DR. Requirement for Tec kinases Rlk and Itk in T cell receptor signaling and immunity. Science. (1999) 284:638–41. 10.1126/science.284.5414.638 10213685

[23] Liu KQ Bunnell SC Gurniak CB Berg LJ. T cell receptor-initiated calcium release is uncoupled from capacitative calcium entry in Itk-deficient T cells. J Exp Med. (1998) 187:1721–7. 10.1084/jem.187.10.1721 9584150 PMC2212298

[24] Atherly LO Lucas JA Felices M Yin CC Reiner SL Berg LJ. The Tec family tyrosine kinases Itk and Rlk regulate the development of conventional CD8^+^ T cells. Immunity. (2006) 25:79–91. 10.1016/j.immuni.2006.05.012 16860759

[25] Broussard C Fleischacker C Horai R Chetana M Venegas AM Sharp LL. Altered development of CD8^+^ T cell lineages in mice deficient for the Tec kinases Itk and Rlk. Immunity. (2006) 25:93–104. 10.1016/j.immuni.2006.05.011 16860760

[26] Prince AL Yin CC Enos ME Felices M Berg LJ. The Tec kinases Itk and Rlk regulate conventional versus innate T-cell development. Immunol Rev. (2009) 228:115–31. 10.1111/j.1600-065X.2008.00746.x 19290924 PMC2669323

[27] Prince AL Watkin LB Yin CC Selin LK Kang J Schwartzberg PL. Innate PLZF^+^CD4^+^ alphabeta T cells develop and expand in the absence of Itk. J Immunol. (2014) 193:673–87. 10.4049/jimmunol.1302058 24928994 PMC4083617

[28] Prince AL Kraus Z Carty SA Ng C Yin CC Jordan MS. Development of innate CD4^+^ and CD8^+^ T cells in Itk-deficient mice is regulated by distinct pathways. J Immunol. (2014) 193:688–99. 10.4049/jimmunol.1302059 24943215 PMC4114307

[29] Hu J Sahu N Walsh E August A. Memory phenotype CD8^+^ T cells with innate function selectively develop in the absence of active Itk. Eur J Immunol. (2007) 37:2892–9. 10.1002/eji.200737311 17724684 PMC2770953

[30] Hu J August A. Naive and innate memory phenotype CD4^+^ T cells have different requirements for active Itk for their development. J Immunol. (2008) 180:6544–52. 10.4049/jimmunol.180.10.6544 18453573 PMC2836934

[31] Huang W Huang F Kannan AK Hu J August A. ITK tunes IL-4-induced development of innate memory CD8^+^ T cells in a gammadelta T and invariant NKT cell-independent manner. J Leukoc Biol. (2014) 96:55–63. 10.1189/jlb.1AB0913-484RR 24620029 PMC4056274

[32] Miller A Wilcox H Lai Z Berg L. Signaling through Itk promotes T helper 2 differentiation via negative regulation of T-bet. Immunity. (2004) 21:67–80. 10.1016/j.immuni.2004.06.009 15345221

[33] Mueller C August A. Attenuation of immunological symptoms of allergic asthma in mice lacking the tyrosine kinase ITK. J Immunol. (2003) 170:5056–63. 10.4049/jimmunol.170.10.5056 12734350

[34] Kannan A Sahu N Mohanan S Mohinta S August A Itk modulates allergic airway inflammation by suppressing IFNγ in naïve CD4^+^ T-cells. J Allergy Clin Immunol. (2013) 132:811–20.e1–5. 10.1016/j.jaci.2013.04.033 23768572 PMC4033298

[35] Kannan A Lee Y Qi Q Huang W Jeong AR Ohnigian S. Allele-sensitive mutant, Itkas, reveals that Itk kinase activity is required for Th1, Th2, Th17, and iNKT-cell cytokine production. Eur J Immunol. (2015) 45:2276–85. 10.1002/eji.201445087 25989458 PMC5730406

[36] Sahu N Venegas AM Jankovic D Mitzner W Gomez-Rodriguez J Cannons JL. Selective expression rather than specific function of Txk and Itk regulate Th1 and Th2 responses. J Immunol. (2008) 181:6125–31. 10.4049/jimmunol.181.9.6125 18941202 PMC2849304

[37] Sahu N Mueller C Fischer A August A. Differential sensitivity to Itk kinase signals for T helper 2 cytokine production and chemokine-mediated migration. J Immunol. (2008) 180:3833–8. 10.4049/jimmunol.180.6.3833 18322190 PMC2913463

[38] Schaeffer EM Yap GS Lewis CM Czar MJ McVicar DW Cheever AW. Mutation of Tec family kinases alters T helper cell differentiation. Nat Immunol. (2001) 2:1183–8. 10.1038/ni734 11702066

[39] Gomez-Rodriguez J Meylan F Handon R Hayes ET Anderson SM Kirby MR. Itk is required for Th9 differentiation via TCR-mediated induction of IL-2 and IRF4. Nat Commun. (2016) 7:10857. 10.1038/ncomms10857 26936133 PMC4782063

[40] Gomez-Rodriguez J Sahu N Handon R Davidson TS Anderson SM Kirby MR. Differential expression of interleukin-17A and−17F is coupled to T cell receptor signaling via inducible T cell kinase. Immunity. (2009) 31:587–97. 10.1016/j.immuni.2009.07.009 19818650 PMC2767186

[41] Gomez-Rodriguez J Wohlfert EA Handon R Meylan F Wu JZ Anderson SM. Itk-mediated integration of T cell receptor and cytokine signaling regulates the balance between Th17 and regulatory T cells. J Exp Med. (2014) 211:529–43. 10.1084/jem.20131459 24534190 PMC3949578

[42] Kannan AK Kim DG August A Bynoe MS. Itk signals promote neuroinflammation by regulating CD4^+^ T-cell activation and trafficking. J Neurosci. (2015) 35:221–33. 10.1523/JNEUROSCI.1957-14.2015 25568116 PMC4287144

[43] Huang W Solouki S Koylass N Zheng SG August A. ITK signalling via the Ras/IRF4 pathway regulates the development and function of Tr1 cells. Nat Commun. (2017) 8:15871. 10.1038/ncomms15871 28635957 PMC5482062

[44] Kannan AK Mohinta S Huang W Huang L Koylass N Appleton JA. T-Bet independent development of IFNgamma secreting natural T helper 1 cell population in the absence of Itk. Sci Rep. (2017) 7:45935. 10.1038/srep45935 28406139 PMC5390256

[45] Czar MJ Debnath J Schaeffer EM Lewis CM Schwartzberg PL. Biochemical and genetic analyses of the Tec kinases Itk and Rlk/Txk. Biochem Soc Trans. (2001) 29:863–7. 10.1042/bst0290863 11709089

[46] Yin CC Cho OH Sylvia KE Narayan K Prince AL Evans JW. The Tec kinase ITK regulates thymic expansion, emigration, and maturation of gammadelta NKT cells. J Immunol. (2013) 190:2659–69. 10.4049/jimmunol.1202531 23378428 PMC3594397

[47] Felices M Yin C Kosaka Y Kang J Berg L Tec kinase Itk in gammadelta T cells is pivotal for controlling IgE production in vivo. Proc Natl Acad Sci USA. (2009) 106:8308–13. 10.1073/pnas.0808459106 19416854 PMC2688853

[48] Qi Q Xia M Hu J Hicks E Iyer A Xiong N Enhanced development of CD4^+^ gammadelta T cells in the absence of Itk results in elevated IgE production. Blood. (2009) 114:564–71. 10.1182/blood-2008-12-196345 19443662 PMC2713465

[49] Xia M Qi Q Jin Y Wiest DL August A Xiong N. Differential roles of IL-2-inducible T cell kinase-mediated TCR signals in tissue-specific localization and maintenance of skin intraepithelial T cells. J Immunol. (2010) 184:6807–14. 10.4049/jimmunol.1000453 20483745 PMC2941197

[50] Lee SH Chang HS Jang AS Park SW Park JS Uh ST. The association of a single-nucleotide polymorphism of the IL-2 inducible T-cell Kinase gene with asthma. Ann Hum Genet. (2011) 75:359–69. 10.1111/j.1469-1809.2010.00637.x 21323647

[51] Ferrara TJ Mueller C Sahu N Ben-Jebria A August A. Reduced airway hyperresponsiveness and tracheal responses during allergic asthma in mice lacking tyrosine kinase inducible T-cell kinase. J Allergy Clin Immunol. (2006) 117:780–6. 10.1016/j.jaci.2005.12.1330 16630934

[52] Alpay F Zare Y Kamalludin MH Huang X Shi X Shook GE. Genome-wide association study of susceptibility to infection by *Mycobacterium avium* subspecies *paratuberculosis* in Holstein cattle. PLoS ONE. (2014) 9:e111704. 10.1371/journal.pone.0111704 25473852 PMC4256300

[53] Kim MJ Wainwright HC Locketz M Bekker LG Walther GB Dittrich C. Caseation of human tuberculosis granulomas correlates with elevated host lipid metabolism. EMBO Mol Med. (2010) 2:258–74. 10.1002/emmm.201000079 20597103 PMC2913288

[54] Seimon TA Kim MJ Blumenthal A Koo J Ehrt S Wainwright H. Induction of ER stress in macrophages of tuberculosis granulomas. PLoS ONE. (2010) 5:e12772. 10.1371/journal.pone.0012772 20856677 PMC2939897

[55] Ritchie ME Phipson B Wu D Hu Y Law CW Shi W. limma powers differential expression analyses for RNA-sequencing and microarray studies. Nucleic Acids Res. (2015) 43:e47. 10.1093/nar/gkv007 25605792 PMC4402510

[56] Luo W Friedman MS Shedden K Hankenson KD Woolf PJ. GAGE: generally applicable gene set enrichment for pathway analysis. BMC Bioinformatics. (2009) 10:161. 10.1186/1471-2105-10-161 19473525 PMC2696452

[57] Subramanian A Tamayo P Mootha VK Mukherjee S Ebert BL Gillette MA. Gene set enrichment analysis: a knowledge-based approach for interpreting genome-wide expression profiles. Proc Natl Acad Sci USA. (2005) 102:15545–50. 10.1073/pnas.0506580102 16199517 PMC1239896

[58] Luo W Brouwer C. Pathview: an R/Bioconductor package for pathway-based data integration and visualization. Bioinformatics. (2013) 29:1830–1. 10.1093/bioinformatics/btt285 23740750 PMC3702256

[59] Carroll P Schreuder LJ Muwanguzi-Karugaba J Wiles S Robertson BD Ripoll J. Sensitive detection of gene expression in mycobacteria under replicating and non-replicating conditions using optimized far-red reporters. PLoS ONE. (2010) 5:e9823. 10.1371/journal.pone.0009823 20352111 PMC2843721

[60] Reece JJ Siracusa MC Southard TL Brayton CF Urban JF Jr Scott AL. Hookworm-induced persistent changes to the immunological environment of the lung. Infect Immun. (2008) 76:3511–24. 10.1128/IAI.00192-08 18505812 PMC2493237

[61] Chackerian AA Alt JM Perera TV Dascher CC Behar SM. Dissemination of *Mycobacterium tuberculosis* is influenced by host factors and precedes the initiation of T-cell immunity. Infect Immun. (2002) 70:4501–9. 10.1128/IAI.70.8.4501-4509.2002 12117962 PMC128141

[62] Feng CG Kaviratne M Rothfuchs AG Cheever A Hieny S Young HA. NK cell-derived IFN-gamma differentially regulates innate resistance and neutrophil response in T cell-deficient hosts infected with Mycobacterium tuberculosis. J Immunol. (2006) 177:7086–93. 10.4049/jimmunol.177.10.7086 17082625

[63] Srivastava S Ernst JD Desvignes L. Beyond macrophages: the diversity of mononuclear cells in tuberculosis. Immunol Rev. (2014) 262:179–92. 10.1111/imr.12217 25319335 PMC4203409

[64] Huang L Nazarova EV Tan S Liu Y Russell DG. Growth of *Mycobacterium tuberculosis in vivo* segregates with host macrophage metabolism and ontogeny. J Exp Med. (2018) 215:1135–52. 10.1084/jem.20172020 29500179 PMC5881470

[65] Mishra BB Lovewell RR Olive AJ Zhang G Wang W Eugenin E. Nitric oxide prevents a pathogen-permissive granulocytic inflammation during tuberculosis. Nat Microbiol. (2017) 2:17072. 10.1038/nmicrobiol.2017.72 28504669 PMC5461879

[66] Dallenga T Repnik U Corleis B Eich J Reimer R Griffiths GW. *M. tuberculosis*-induced necrosis of infected neutrophils promotes bacterial growth following phagocytosis by macrophages. Cell Host Microbe. (2017) 22:519–30.e3. 10.1016/j.chom.2017.09.003 29024644

[67] Gopal R Monin L Slight S Uche U Blanchard E Fallert Junecko BA. Unexpected role for IL-17 in protective immunity against hypervirulent *Mycobacterium tuberculosis* HN878 infection. PLoS Pathog. (2014) 10:e1004099. 10.1371/journal.ppat.1004099 24831696 PMC4022785

[68] Lockhart E Green AM Flynn JL. IL-17 production is dominated by gammadelta T cells rather than CD4 T cells during *Mycobacterium tuberculosis* infection. J Immunol. (2006) 177:4662–9. 10.4049/jimmunol.177.7.4662 16982905

[69] Horvath CN Shaler CR Jeyanathan M Zganiacz A Xing Z. Mechanisms of delayed anti-tuberculosis protection in the lung of parenteral BCG-vaccinated hosts: a critical role of airway luminal T cells. Mucosal Immunol. (2012) 5:420–31. 10.1038/mi.2012.19 22453678

[70] Segueni N Tritto E Bourigault ML Rose S Erard F Le Bert M. Controlled *Mycobacterium tuberculosis* infection in mice under treatment with anti-IL-17A or IL-17F antibodies, in contrast to TNFalpha neutralization. Sci Rep. (2016) 6:36923. 10.1038/srep36923 27853279 PMC5113257

[71] Segueni N Benmerzoug S Rose S Gauthier A Bourigault ML Reverchon F. Innate myeloid cell TNFR1 mediates first line defence against primary *Mycobacterium tuberculosis* infection. Sci Rep. (2016) 6:22454. 10.1038/srep22454 26931771 PMC4773807

[72] Benmerzoug S Bounab B Rose S Gosset D Biet F Cochard T. Sterile lung inflammation induced by silica exacerbates *Mycobacterium tuberculosis* infection via STING-dependent type 2 immunity. Cell Rep. (2019) 27:2649–64.e5. 10.1016/j.celrep.2019.04.110 31141689

[73] Samstein M Schreiber HA Leiner IM Susac B Glickman MS Pamer EG. Essential yet limited role for CCR2(+) inflammatory monocytes during *Mycobacterium tuberculosis*-specific T cell priming. Elife. (2013) 2:e01086. 10.7554/eLife.01086.013 24220507 PMC3820971

[74] Antonelli LR Gigliotti Rothfuchs A Goncalves R Roffe E Cheever AW Bafica A. Intranasal Poly-IC treatment exacerbates tuberculosis in mice through the pulmonary recruitment of a pathogen-permissive monocyte/macrophage population. J Clin Invest. (2010) 120:1674–82. 10.1172/JCI40817 20389020 PMC2860920

[75] La Manna MP Orlando V Dieli F Di Carlo P Cascio A Cuzzi G. Quantitative and qualitative profiles of circulating monocytes may help identifying tuberculosis infection and disease stages. PLoS ONE. (2017) 12:e0171358. 10.1371/journal.pone.0171358 28208160 PMC5313257

[76] Cohen SB Gern BH Delahaye JL Adams KN Plumlee CR Winkler JK. Alveolar macrophages provide an early *Mycobacterium tuberculosis* niche and initiate dissemination. Cell Host Microbe. (2018) 24:439–46.e4. 10.1016/j.chom.2018.08.001 30146391 PMC6152889

[77] Huang W Morales JL Gazivoda VP August A. Nonreceptor tyrosine kinases ITK and BTK negatively regulate mast cell proinflammatory responses to lipopolysaccharide. J Allergy Clin Immunol. (2016) 137:1197–205. 10.1016/j.jaci.2015.08.056 26581914 PMC5730405

[78] Iyer AS Morales JL Huang W Ojo F Ning G Wills E. Absence of Tec family kinases interleukin-2 inducible T cell kinase (Itk) and Bruton's tyrosine kinase (Btk) severely impairs Fc epsilonRI-dependent mast cell responses. J Biol Chem. (2011) 286:9503–13. 10.1074/jbc.M110.165613 21212279 PMC3059023

[79] Esin S Shigematsu M Nagai S Eklund A Wigzell H Grunewald J. Different percentages of peripheral blood gamma delta + T cells in healthy individuals from different areas of the world. Scand J Immunol. (1996) 43:593–6. 10.1046/j.1365-3083.1996.d01-79.x 8633219

[80] Huang W Jeong AR Kannan AK Huang L August A. IL-2-inducible T cell kinase tunes T regulatory cell development and is required for suppressive function. J Immunol. (2014) 193:2267–72. 10.4049/jimmunol.1400968 25063868 PMC4352551

[81] Sagiv-Barfi I Kohrt HE Czerwinski DK Ng PP Chang BY Levy R. Therapeutic antitumor immunity by checkpoint blockade is enhanced by ibrutinib, an inhibitor of both BTK and ITK. Proc Natl Acad Sci USA. (2015) 112:E966–72. 10.1073/pnas.1500712112 25730880 PMC4352777

