# Supplementary figures and images for: Interleukin-2-Inducible T-Cell Kinase Deficiency Impairs Early Pulmonary Protection Against Mycobacterium tuberculosis Infection
Source: Front Immunol. 2020 Jan 24;10:3103. doi: 10.3389/fimmu.2019.03103 (PMC6993117; doi:10.3389/fimmu.2019.03103)

## Slide 1
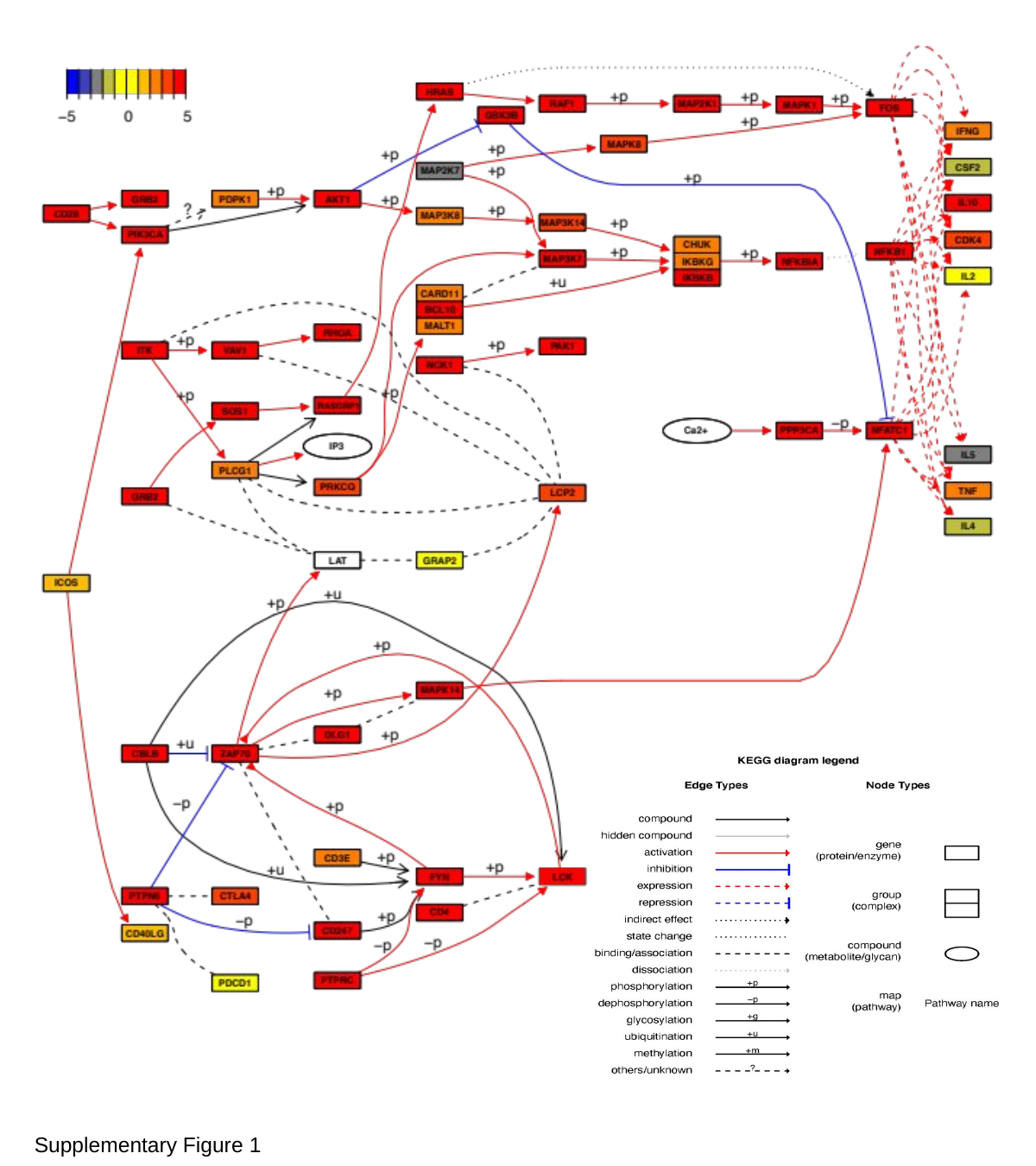

Supplementary Figure 1

Supplement: Supplementary Figure 1 — Gene enrichment profile of the TCR/ITK signaling in human lungs with active TB. Genes in the TCR signaling pathways were rendered with their positions and connections in the pathway, with colored codes indicating the log2-fold change comparing levels of gene expression in caseous to those in normal tissues. Red indicates upregulation in caseous samples while blue indicates downregulation. Note that ITK is one of the central components of the TCR signaling pathway. [file Presentation_1.pptx]
